# Magnetic Fluid‐Driven Vine Robots for Minimally Invasive Tissue Biopsy Sampling

**DOI:** 10.1002/aisy.202400827

**Published:** 2025-03-27

**Authors:** Joshua Davy, Thomas P. Dean, Nikita J. Greenidge, Benjamin Calmé, Peter Lloyd, James H. Chandler, Pietro Valdastri

**Affiliations:** ^1^ STORM Lab School of Electronic and Electrical Engineering University of Leeds Woodhouse Leeds LS2 9JT UK

**Keywords:** biopsies, endoluminal navigation, magnetic manipulations, surgical robotics, vine robots

## Abstract

There is a growing need for precise, minimally invasive biopsy techniques that reduce patient discomfort, improve sampling accuracy in hard‐to‐reach areas, and minimize tissue damage. Vine robots, a type of continuum robot, offer a promising solution with their unique ability to evert, allowing them to navigate complex environments while reducing friction. This article presents a novel vine robot design powered by magnetic fluid. The fluid drives both vine growth through pressurization and enables precise steering and manipulation using external magnetic fields. Unlike previous designs, the robot's high magnetic volume ensures precise control even under pressure, while maintaining a fully soft structure. This allows for controlled needle movements during biopsies. Additionally, the robot achieves passive stabilization by pressing against surrounding walls. This stabilization, combined with magnetic forces, can exert up to 1.26 N of insertion force at the tip, enabling effective tissue penetration. Experiments are conducted with a 5 mm diameter, 145 mm long magnetic fluid‐driven vine robot, demonstrating movement in free space, through narrow constrictions, and within phantoms modeled after human bronchial anatomy. These results pave the way for the robot's potential application in minimally invasive surgeries, particularly in difficult‐to‐access areas of the body.

## Graphical abstract

This study introduces a magnetic fluid‐powered vine robot for minimally invasive biopsies. The robot uses fluid pressurization for locomotion and external magnetic fields for precise steering. Its soft structure and high magnetic volume ensure controlled movements and stabilization, enabling effective tissue penetration. Experiments demonstrate its navigation through complex environments, showcasing potential for surgeries in hard‐to‐reach areas like human bronchial anatomy.

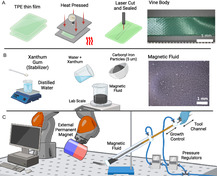

## 1 Introduction

Any decision to perform an endoscopic or catheter‐enabled intervention must be weighed against the risk of procedural complications.^[^ ^1^ ^]^ Standard endoscopic instruments are formed of hard bodies (relative to the anatomy through which they will travel) and thus carry the potential for traumatic tissue interaction. These instruments are typically controlled by internal tendons allowing limited bending focused purely at the tip and their insertion through the anatomy can be limited by frictional forces with surrounding tissues.^[^ ^2^ ^]^ Such tools are often characterized by low dexterity, limited control, and large diameter.^[^ ^3^ ^]^ These challenges motivate the design of new robotic instruments^[^ ^3^, ^4^ ^]^ one of which is the everting catheter.^[^ ^5^, ^6^, ^7^ ^]^ These devices utilize an unraveling material which, instead of translating through a lumen, locomote via tip‐centered growth. This form of locomotion minimizes potentially traumatic tissue interaction and focuses the forces of insertion at the distal rather than proximal end of the device.^[^ ^6^ ^]^ Recently, such robots integrated with robotic steering methods such as pneumatic artificial muscles have been designated as “soft growing robots” or “vine robots” (so named due to their visual and behavioral similarity to the climbing plant) and have been explored for a wide range of applications including military, civil engineering, and medical.^[^ ^8^, ^9^ ^]^

In the medical domain, these vine robots form part of a wider group of soft robotic systems where it is hoped that softer materials can minimize stress on surrounding tissue during physical interaction.^[^ ^10^ ^]^ For target applications in the navigation of tight lumens, a longstanding goal has been the miniaturization of such devices. With decreased vine diameter comes a reduced tip cross‐sectional area and therefore the internal pressure required to evert the material increases.^[^ ^11^ ^]^ Further, as with any robotic system, miniaturization creates challenges due to the difficulty of small‐scale manufacturing and the inevitable reduction in usable actuating force.

Nonsteered everting catheters have long existed, with some approved for clinical use.^[^ ^5^ ^]^ Given their soft conforming bodies, they are capable of passively adapting to anatomical paths requiring no active steering mechanism.^[^ ^12^ ^]^ A more recent example would be VINE (Vascular Internal Navigation by Extension), an everting catheter 3.5 mm in diameter designed for endovascular surgery. This device, instead of utilizing an internal steering mechanism, is preshaped based on the target anatomical structure and grows in a predetermined direction.^[^ ^13^ ^]^ This approach is capable of high levels of curvature and miniaturization, but the design is predetermined and therefore must be manufactured to the specific anatomy of interest. Without this procedure‐specific approach, natural bifurcations, such as those commonly found in the anatomy, require active steering mechanisms to navigate in a deterministic manner. Away from the medical domain, Hawkes et al. introduced pneumatic artificial muscles for steering of 25 mm diameter vine robots.^[^ ^8^ ^]^ These inflatable pouches on the side of the robot enable steering via pressurization at the base. However, the complexity in the fabrication of these pouches has likely prevented it from being applied at a smaller scale.

The Mammobot platform is one such miniaturized vine robot designed for navigation of bifurcations in the mammary glands.^[^ ^7^ ^]^ This device utilizes an internal steerable catheter to bend the vine body and has been demonstrated at a 2.9 mm‐diameter scale. The tendon‐driven catheter is limited in its dexterity, but is capable of steering the vine through small bending angles. This design could be considered more of an everting sheath rather than a self‐propelling vine robot, as its locomotion is determined by the pushing of the stiff internal tool rather than internal pressurization. This stiff tool provides the steering mechanism for the robot but sacrifices the overall softness of the robot body and, with it, limits bending curvature and overall conformity. Furthermore, a key advantage of everting bodies is the creation of locomotion force at their tip. With this approach, force is still applied from the proximal end as a conventional catheter would do.

This need for precise application of forces has given rise to the use of magnetics for robotic actuation. Here, magnetic elements within the robot body can be manipulated via externally applied magnetic fields. This avoids complex internal mechanisms while retaining the ability to induce precise forces on the instrument. This forms a wireless actuation methodology where the induced torques and forces are proportional to both the volume of internal magnetic material and the magnitude of the applied magnetic field and gradients. Magnetic instruments have been designed both from hard permanent magnet elements or via integrating magnetic micropowders (such as NdFeB) into silicone elastomers.^[^ ^2^, ^14^ ^]^ The latter retains a fully soft structure throughout the device. More recently, soft robot designs have utilized magnetic fluids. These can be free‐flowing magnetic fluids capable of controlling both their movement and geometry or constrained magnetic fluids placed within soft bodies.^[^ ^15^, ^16^ ^]^ Leon‐Rodriguez et al. presented an untethered everting design inspired by the motion of *Amoeba* bacteria actuated by an internal magnetic fluid.^[^ ^17^ ^]^ This robot is pulled by magnetic field gradients, moving without relative shear and therefore low friction all while retaining a fully soft body.

In our previous work, we introduced an 8 mm diameter vine structure exhibiting a magnetic skin upon the vine wall which directed the growth of the robot through various vessels.^[^ ^18^ ^]^ To enable successful eversion, vines require a sufficiently flexible, and therefore thin, wall structure. Due to this constraint, and the integration of magnetic material into the walls, the magnetic volume of this device was limited meaning the induced force and torque on the system was low. This system therefore required low separation between the external permanent magnet (EPM) and the robot for successful navigation and was limited in terms of further miniaturization. Indeed, the low induced magnetic force and torques meant that deformation while the vine robot was pressurized was minimal except in the case of immediate EPM proximity. A strategy of alternate growing and steering was thus utilized making complex navigations a challenge which required large magnetic fields. This effect would be exacerbated in the presence of internal tools such as a biopsy needle, further augmenting the effective stiffness of the body and making steered navigation near impossible. We have previously explored hard magnetic tips featuring permanent magnets for vine steering at the 25 mm diameter scale.^[^ ^19^ ^]^ This provides a large magnetic volume but sacrifices the soft body of the vine robot, leaving a hard tip. This can introduce some degree of shear translation at the tip and can therefore potentially still lead to traumatic tissue interaction, particularly in small diameter lumen. Further, for the case of tissue biopsy, where applying large forces is needed, the conformity of entire soft bodies provides a point of stabilization, which can be leveraged to provide greater reaction forces.^[^ ^20^ ^]^ **Table** **1** summarizes key approaches to vine robot steering control as well as target application.

**Table 1:** Comparison of notable steering mechanisms and their applications.

Authors	Steering mechanism	Diameter [mm]	Body	Application
Hawkes et al.^[^ ^8^ ^]^	Pneumatic artificial muscles	25	Soft	First exploration of steerable vine robots
Berthret‐Rayne et al.^[^ ^7^ ^]^	Internal hard tendon	2.9	Hard, stiff internal tendon	Navigation of the mammary glands
Li et al.^[^ ^13^ ^]^	None (preshaped to anatomical structure)	3.5–5	Soft	Endovascular surgery
Kim et al.^[^ ^19^ ^]^	Permanent magnet at the tip	25	Hard tip but soft body	Robotic colonoscopy
Davy et al.^[^ ^18^ ^]^	Magnetic skin	8	Soft	Navigation of upper bronchial tree
This Work	Internal magnetic fluid	4–7	Soft	Navigation of deep bronchial tree and biopsy of soft tissue

A biopsy is one of the most common endoluminal procedures, involving the extraction of a small tissue sample for histological analysis to differentiate between healthy and cancerous tissues. It is also one of the most challenging to achieve with soft systems, while being among the most demanding in terms of force transmission. Recent research has introduced soft robotic platforms with origami‐inspired architectures that allow needle deployment, demonstrating promising results.^[^ ^20^ ^]^ However, these designs remain limited as they require the needle to be exposed during the robot's deployment and are also constrained in their potential miniaturization. Current approaches are also constrained in terms of force transmission with a maximum of 1 N^[^ ^20^, ^21^ ^]^ whereas the required force is closer to 2 N in the case of stiffer tissues.^[^ ^22^ ^]^

In this study, we consider vine robots driven by a pressurized magnetic fluid. By utilizing a magnetic fluid, we form a vine robot with an entirely soft body but also high magnetic volume, enabling growth through hydraulic pressure and steering under external magnetic fields. We show this design to be highly miniaturizable and explore designs in the 4–7 mm‐diameter range. We show how these bodies are capable of conforming to the anatomy and are not limited by friction when navigating a lumen due to the entirely soft body of the robot. This device is capable of high curvature steering in any plane based on the positioning of the EPM. We chose tissue biopsy in the bronchial tree as a clinical case study and, by integrating an endoscopic camera and biopsy needle into our vine robot, demonstrate camera‐assisted navigation within a bronchial tree phantom and the puncturing of soft tissue with a needle, leading the way toward clinical application.

In summary, we make the following contributions: 1) the introduction of a uniquely miniaturized vine robot capable of creating high body forces for steering via magnetic fields but crucially retaining robot body softness while producing forces at the tip greater than 1 N; 2) an evaluation of this robot at various diameters and its ability to navigate tight lumen; and 3) a preclinical study of the application of this unique robot to tissue‐biopsy procedures in the lungs through a clinically relevant phantom.

## 2 Fundamentals

### 2.1 Eversion

**Figure 1:**
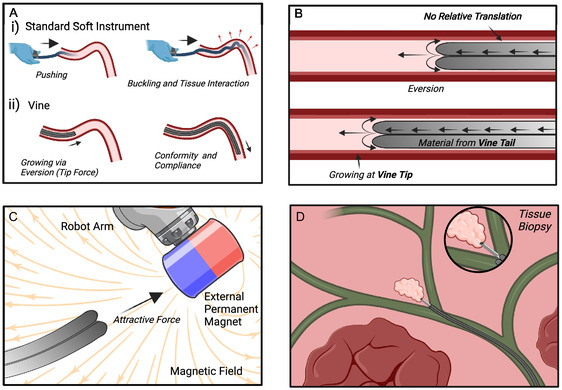
A) A traditional soft catheter is prone to buckling and creates high forces on the surrounding vessels. A vine robot concentrates its force at the tip and moves in a shear‐free manner. B) Vines locomote via everting; the inner tail moves forward due to pressure and everts at the tip. The everted material remains static as the vine extends its length. C) Magnetic fluid‐driven vines can be steered using external permanent magnets (EPMs) to induce forces on the body. D) Magnetic fluid driven vines can be highly miniaturized and enable navigation deep within the anatomy.

Vine robots took a simple form. The outer body was that of a thin‐walled flexible tube, inverted into itself creating a double layer that was enclosed at one end (hereby referred to as the vine tip (see **Figure** **1**B). Pressurization of this internal cavity caused the internal material to be expelled at the tip, leading to elongation of the body in a process known as vine eversion or growth. Constraining the internal material (the vine tail) and management of its release allowed the eversion process to be regulated. As the vine body was growing from the tip rather than translating relative to the environment, external friction was negated, with the only friction in the system being within the vine body between everted and inverted material.

Due to this frictionless shear‐free navigation, vine robots were of great interest for applications in endoluminal surgery.^[^ ^7^, ^13^, ^18^ ^]^ Traditional instruments were often formed of hard bodies and locomote via pushing from their proximal end (Figure 1A). In contrast, vines were formed of entirely soft bodies and locomoted via growth rather than translation. This had the potential to reduce potentially traumatic tissue interaction with vessels within the body and could enable navigation to areas of the anatomy that would traditionally be considered unreachable or too dangerous to access.^[^ ^18^ ^]^ Further, the inherent channel within the vine tail could be utilized for irrigation, or incorporating standard endoscopic tools such as a biopsy needle, allowing useful access deep within the human anatomy.

### 2.2 Magnetic Fluids

Vine robot designs had been proposed which utilized both pneumatic and hydraulic pressurization for eversion. In this work, we propose a vine robot actuated via an internal magnetic liquid. Here, pressurization led to vine growth, and steering of the robot across bifurcations was achieved through magnetic attraction. Forces could be applied directly to the magnetic fluid via the application of external magnetic fields. Magnetic fluids typically took the form of dispersed magnetic particles in a carrier liquid and could be categorized into ferrofluids and magnetorheological fluids.^[^ ^23^ ^]^ The former was commonly formed from magnetic nanoparticles in permanent suspension, often in a highly toxic petroleum‐based carrier liquid. Alternatively, magnetorheological fluids were based on much larger particles in the micron range. The carrier fluid was typically water or oil and was typically of a greater magnetic volume (20–40%) than ferrofluids (10–20%). However, due to their large particle size and concentration, the suspension was only temporary. Typically, these fluids utilized a thixotropic agent to stabilize the fluid and reduce sedimentation.^[^ ^24^ ^]^ Magnetorheological fluids were named as they exhibited the magnetorheological effect. This effect was characterized by the dependence of the material's viscosity on the applied magnetic field. This was due to the alignment of particles once magnetized, forming chains within the material, and under certain conditions, this fluid could even become a viscoelastic solid. For magnetic fluids, the ability of the suspended magnetic particles to freely rotate meant that they aligned with the direction of applied field nearly instantaneously (constrained only by viscosity). Consequently, for magnetic fluids, no material torque would be present and any actuation would be purely gradient‐force based. In the case of manipulation with external permanent magnets, this force would be directed toward the poles of the EPM.

**Figure 2:**
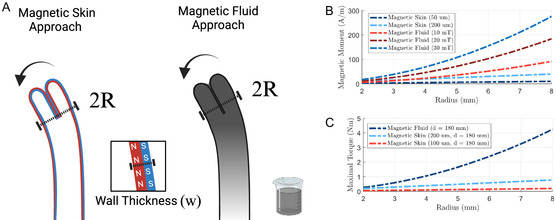
A) Diagram demonstrating the significant increase in magnetic volume achieved with the magnetic fluid approach of this work, rather than the magnetic skin approach of our previous work.^[^ ^18^ ^]^ B) Graph comparing magnetic skin to magnetic fluid under various fields and wall thickness for a 50 mm length vine (see Appendix for details). C) Graph showing maximal induced torque under rigid body assumptions between the two approaches given an EPM of diameter 101 mm, length 101 mm (*R*_e_ = 1.44 T) was positioned 150 mm away.

As mentioned previously, our earlier work considered vine robots with magnetic skin.^[^ ^18^ ^]^ This utilized hard magnetic NdFeB microparticles which stored a much higher remanent magnetization under typical fields. However, the inherent requirement for vines to be formed of thin‐wall materials limited the overall magnetic volume. In **Figure** **2**, we compare the approach of magnetic fluid with that of magnetic skin for a 50 mm length vine. Full details of the assumptions made are provided in the Appendix. It can be observed that the magnetic fluid approach produced a significantly higher magnetic moment across these small scales and resulted in much higher actuation torques while retaining the soft structure, motivating this new design of miniaturized magnetic vine robot.

### 2.3 Functionalization

The use of tools such as biopsy needles in vine robots presented a significant challenge. Due to the everting structure, no part of the material body could be defined as the permanent tip and therefore no direct attachment point was available. This is further exacerbated by the fact that the internal material moves at twice the speed of the tip. This leads to the so‐called spitting problem in which anything placed within the vine's inner channel is ejected as the vine everts. Furthermore, the pressurization of the vine body contracted onto this inner channel, preventing the free movement of tools. Therefore, any device needing to maintain position at the tip of the robot during growth must somehow be synchronized with the eversion process. This could be achieved by a duty cycling of pressurization, growth, depressurization, and then tool retraction.^[^ ^7^ ^]^ Given the vine robots’ ability to navigate constricted channels without friction, this contact can be exploited as a point (or area) of stabilization for applying large forces (such as those required for tissue penetration) at the robot tip. This however does require the channel to be smaller than the vine robot's nominal diameter.

**Figure 3:**
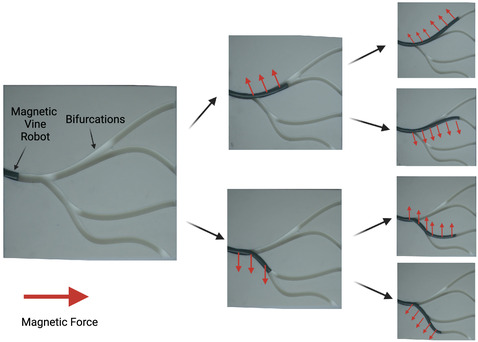
Figure showing navigation of tight bifurcations. Movement of the external EPM creates a net magnetic force on the entire robot body. As the vine robot everts, the direction of this growth can be controlled by manipulating the magnetic field with the EPM. Despite this force being experienced across the whole body, the constraint of the lumen permits only the tip to be manipulated.

With the combination of fluidic actuation for growth and an internal magnetic fluid enabling steering, magnetic‐fluid‐driven vine robots retained the simplicity of vine robot form, exhibited an entirely soft robot body, and gave rise to the potential for a highly miniaturized design. Given our requirement for the use of tools with the vine robot, the relatively high forces and torques of the magnetic fluid approach solved this, preserving the soft body but giving the operator a high degree of control, all while providing an intuitive control system via positioning of an external magnet. The magnetic fluid within the robot's structure created forces under a gradient magnetic field that acted on the entire body, not just the tip. “Body steering” methods were well studied in pneumatic vine robots and had been demonstrated for navigating various complex environments.^[^ ^25^ ^]^ In constrained environments such as bifurcations and narrow lumens, the robot's body was constrained with little freedom for movement allowing tip‐focused navigation via manipulation of the magnetic field (see **Figure** **3**).

**Figure 4:**
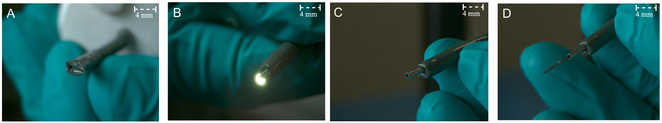
A) A vine robot of 4 mm diameter filled with magnetic fluid. B) An optical fiber inserted into the vine. C) Retracted biopsy needle. D) Extended biopsy needle.

In the following sections, we will develop a methodology for evaluating the potential of these robots for navigation within the anatomy via endoscopic camera for positioning a biopsy needle for target applications in minimally invasive surgery (see **Figure** 1 and **4**).

## 3 Experimental Section

### 3.1 Vine Robot Body

**Figure 5:**
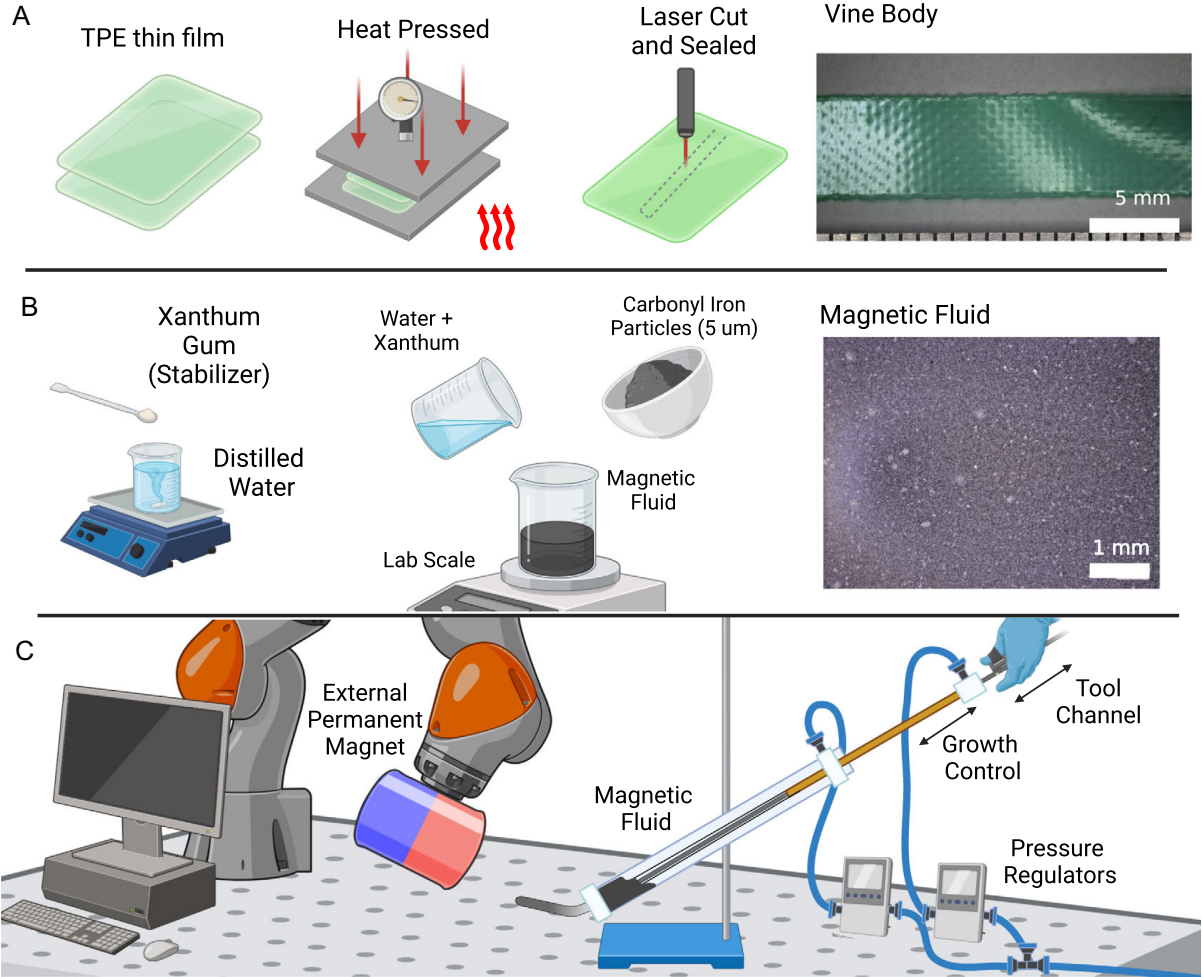
A) Fabrication of the vine body. Two layers of thermoplastic‐elastomer are layered and are softly heat pressed to form a temporary bond. The laser cutter seals and cuts the vine body. B) Preparation of the magnetic fluid. Xanthum gum acts as a stabilizer to maintain a suspension of the iron particles. C) The vine robot is steered using an EPM mounted to a robotic manipulator. The vine's growth is controlled via insertion of a TPU tube that also provides a tool channel.

The vine robot body must be formed of a thin‐wall and sufficiently flexible tube in order for successful growth under eversion. Based on previous work, we selected a 38 μm‐thick thermoplastic‐elastomer (TPE) film (Stretchlon 200, Airtech Advanced Materials, UK) as our vine material. For these thin geometries, this material (*E* = 10 MPa) is flexible but can be considered of low extensibility at our operating pressures. The low melting point of the material allows it to be easily sealed with heat. We utilize a laser‐welding technique to form the vine into a layflat tube of length 290 mm.^[^ ^26^ ^]^ This is achieved first by soft sealing two layers of the material at 100 °C on a heat press (Combo Heat Press, Creworks, USA) for 2 min to bring the two layers into close contact. The pressure of the heat press was manually tuned to allow the layers to be adhered but separable by hand. After this, the tube was formed using an optical laser cutter (Creality Falcon 2 Pro 22 W, Creality, China) to simultaneously seal and cut (see **Figure** **5**A). The width of the layflat tube corresponds to *π*/2 of the inflated body diameter. The parameters utilized for the laser cutter were determined empirically. A speed of 2733 mm min^−1^ with a laser power of 50% was found to give a seal that was not prone to leakage. Once cut, the vine body was then inflated and checked for leaks. The completed vine is of length 145 mm. Once pressurized, due to the elasticity of the vine material, the diameter will increase slightly. However, this is shown to be less than 1 mm under typical operating pressures (see Appendix).

### 3.2 Magnetic Fluid

Due to the toxicity and relatively low magnetic volume of commercial ferrofluids, we utilized an iron–water suspension for the magnetic growing fluid within the robot. The fluid was manufactured as outlined in McDonald et al.^[^ ^24^ ^]^ This fluid was selected due to its simplicity and the intrinsic biocompability of its constituent parts. First, 0.2% by weight xanthan gum was mixed with water, and then 23% by volume carbonyl iron particles (1–6 μm mean particle size, 98% purity, GF42083210, Goodfellow) were added. The mixture was then mixed at 1600 RPM for 2 min. The xanthan gum acts as a stabilizer, prolonging the suspension of the particles (see Figure 5B). Through experimentation, it was found the fluid remained in an adequate suspension for up to 12 h, after which point settling of the iron particles could be observed (see Appendix A3). Repeating the mixing stage, however, again dispersed the particles. This will affect the lifetime in which the robot can be used before the magnetic fluid needs to be replaced. Carbonyl iron particles were selected due to their high purity and iron's intrinsic high magnetic susceptibility.

### 3.3 Hardware

For creating the external field for magnetic manipulation, a robotic manipulator (iiwa14 LBR, Kuka, Germany) was used with a large EPM (101.6 mm diameter, 101.6 mm length, N52 NdFeB) as an end‐effector (see Figure 5C). The pose of the EPM was controlled in end‐effector space via an external gamepad enabling teleoperated control by the user. The vine body was attached to a clear tube where pressure could be applied and was inverted with the tail attached to a smaller internal tube (see Figure 5C). This internal tube manages the growth of the vine and also permits tool insertion into the vine body. Magnetic fluid was inserted into the outer tube filling the vine. Metallic components were deliberately avoided in the base mechanism to prevent influences on the magnetic field. Given the relatively weak field produced by the magnetic fluid reservoir itself, this has a negligible effect on the vine robot's performance. An electronic pressure regulator (ITV1010‐211BL, SMC, USA) was used to pressurize the air above the magnetic fluid in order to control the vine pressure. A secondary regulator was also used to equalize the pressure in the internal tool channel. This equalizing pressure reduces friction within the tool channel allowing the tool to be inserted and retracted from the vine.

## 4 Results

### 4.1 Growing and Steering

**Figure 6:**
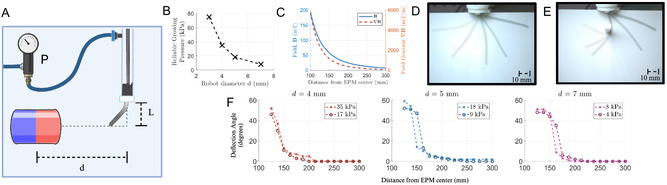
A) Experimental setup for analysis of deflection of the vine under varying external fields. The distance between EPM and vine base *d* is varied causing a deflection of the vine body. B) Vine diameter versus reliable growing pressure. The high pressure of the 3 mm vine made it prone to bursting. C) The magnetic fields and field gradients produced by the EPM (|m| = 970.1 A m^−1^) under dipole assumptions. D) Deflection of the vine on a planar surface with an external magnet. E) Deflection of the vine in the presence of an obstacle. F) Vine deflection versus distance from the EPM at the reliable growing pressure and half the reliable growing pressure.

Four vine robots were produced with 3, 4, 5, and 7 mm diameters respectively. Each was inverted and the internal pressure increased until the vine began to evert. This was repeated three times until a reliable growing pressure for each diameter vine was established (see **Figure** **6**B). As can be observed, smaller diameter vines require larger pressures to evert. This can be attributed to their smaller tip surface area and greater friction within the body. The high pressures required for the growth of the 3 mm vine (75 kPa) provoked bursting and therefore this diameter was not further analyzed.

Each vine was analyzed for its ability to deflect with the external magnet. The vines were placed vertically and the EPM was introduced causing deflection (see Figure 6A). The vines were pressurized to their respective growing pressure and grown to a length *L* = 50 mm while the EPM distance *d* was varied. The EPM was vertically positioned so the tip of the vine aligned with the central axis of the EPM at rest. This process was repeated at half the growing pressure. For each measurement, we performed a total of three experimental repeats. It can be observed that pressure has little effect on deflection. Indeed, deflection is minimal up to a critical distance at which the vine deflects sharply toward the EPM. This critical distance was ≈160 mm for all vines corresponding to a magnetic field of magnitude of 56 mT and field gradient of 1134 mT m^−1^. The maximal achieved deflection for each of the vines against gravity was 52, 59, and 51° respectively (see Figure 6F).

Figure 6D shows the deflection of a 4 mm vine with an external magnet. Here the vine lays flat on a surface. It can be observed that without gravity acting directly against the deflection, vine bending is much more controllable and a variety of deformations can be achieved demonstrating deflection up to 70°. Further, Figure 6E shows how the vine can still be deflected in the presence of an obstacle, the soft body allowing it to conform to the environment (see Video S1, Supporting Information).

### 4.2 Squeezing

**Figure 7:**
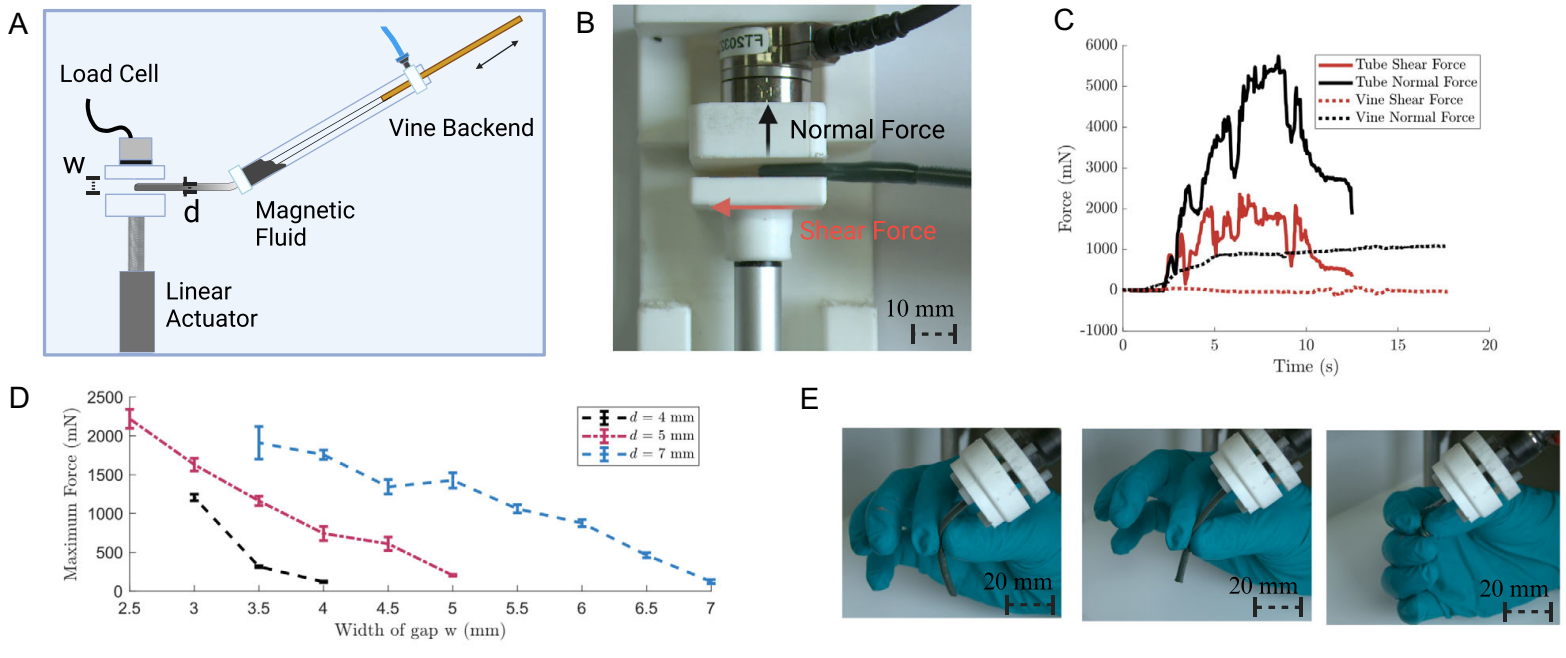
A) Schematic experimental setup for evaluation of forces during vine locomotion. B) Real experimental setup showing directions of normal and shear forces. C) Comparison of insertion of TPU tube with vine robot (both 5 mm diameter). The vine robot produces near zero shear forces and a reduction of normal force of 72.54%. D) Maximum force of insertion versus width of gap navigated for 4, 5, and 7 mm diameter. E) Conformity and reliance of the robot body to external forces.

A key characteristic of vine robots is their ability to squeeze through gaps smaller than their diameter. Our magnetic fluid‐driven vine with its entirely soft body retains this property. In the clinical case, it is important that the vine can navigate through vessels without the application of large forces on surrounding walls. To explore this, we grew vines through a variable width gap of length 25 mm. This gap width was varied via a linear actuator (see **Figure** **7**A,B 6). We quantified the forces applied in the shear and normal directions utilizing a six‐axis force sensor (Nano17, ATI Industrial Automation, USA) repeating three times. Figure 7D shows the maximum applied normal force for 4, 5, and 7 mm vines for varying gap widths at their respective established growing pressures. Where the vine was not able to squeeze through the gap, the data is not presented. Notably, the 5 mm vine is capable of squeezing through the smallest gap of 2.5 mm outperforming the 4 mm vine in this metric. This can be attributed to the fact that although friction will be greater in the larger diameter vine (due to the increase in tail material), the surface area of the tip is larger and therefore a larger growing force is produced.

In Figure 7C, we compare the vine's shear and normal forces to the insertion of a soft tube (O.D 4 mm I.D 3 mm, TPU) with a 4 mm magnetically driven vine through a 3 mm gap. Over three repeats, we consider the average peak force produced. As would be anticipated, the vine creates near zero shear forces (10.3 mN) under insertion, compared to the average peak of 1.04 N by the tube. The vine does produce some normal forces (2.87 N); however, they are considerably less than the tube insertion (7.88 N).

### 4.3 Biopsy Forces

**Figure 8:**
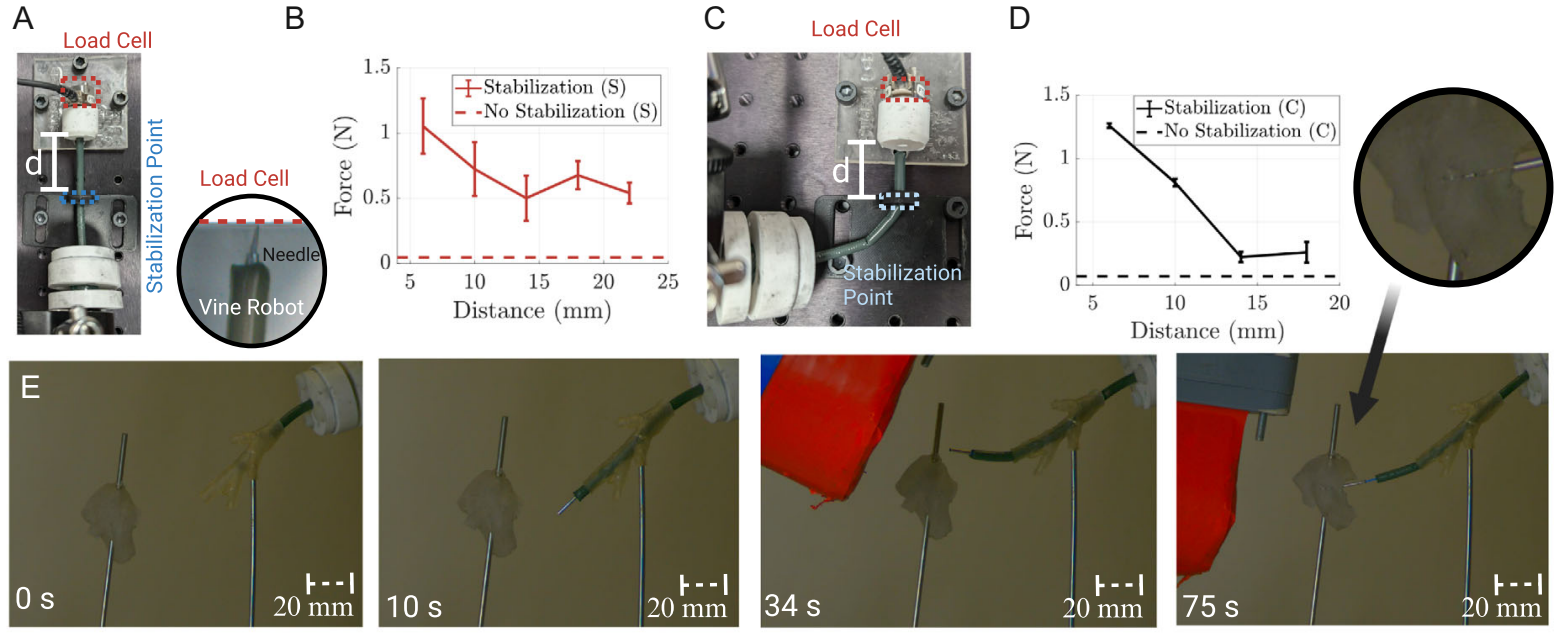
Experimental setup to quantify maximal force output of the biopsy needle with respect to stabilization of the robot. A) Scenario S (Straight). The stabilization point and load cell are parallel to the robot base. B) Maximal force output before buckling with varying distance *d* with and without stabilization for Scenario S. C) Scenario C (corner). The stabilization point and load cell are orthogonal with the robot base. D) Maximal force output before buckling with varying distance *d* with and without stabilization for Scenario C. E) Manipulation of the vine with the biopsy needle inserted through the tool channel via movement of the EPM. At 75 s, the needle is extended to penetrate a piece of a soft tissue phantom.

When the vine is in contact with its surroundings it can stabilize via anatomical contact and exploit this stability to apply large tip forces. We consider the application of tissue biopsy procedures. A biopsy tool formed of a hollow needle tip (20 G, 0.91 mm diameter) was attached to a modified thin‐wire endoscopic tool (FG‐25d retrieval tool, Olympus, Japan) and inserted into the vine robot. We evaluated the vine's ability to apply a force at the tip, given its grown length. A load cell was positioned at the tip of the vine, and the tool was inserted into the vine robot. The maximum force was recorded just before the robot began to buckle at its base. A stabilization point was added between the vine and the load cell (see **Figure** **8**A,C). The vine had a diameter of 5 mm, while the circular stabilization point had a diameter of 4 mm. The robot's grown length was 75 mm and the internal vine pressure was 35 kPa. Two scenarios were examined: in the first, the vine was aligned parallel to the load cell (Figure 8A, Scenario S), and in the second, the vine was oriented perpendicular to the load cell (Figure 8C, Scenario C). The maximal force was recorded for varying distance *d* between the stabilization point and the load cell over three repeats. Figure 8B,C plots the maximal force with respect to *d*. Finally, in each of the scenarios, the vine was deflated to represent the case of no stabilization on surrounding anatomy. It can be observed in this case of no stabilization the force output was significantly less than with the vine acting to stabilize the tool. The maximal force output was 1.05 and 1.26 N in each of the scenarios respectively. This is dependent on the close proximity between the stabilization point and applied force. This represents the need for stabilization close to the target tissue in the case of high tissue stiffness.

In Figure 8E, we demonstrate the ability to steer the vine robot with the inserted needle via movement of the EPM. At 75 s, the vine robot is positioned accordingly and the soft tissue phantom is penetrated (see Video S2, Supporting Information).

### 4.4 Navigation

**Figure 9:**
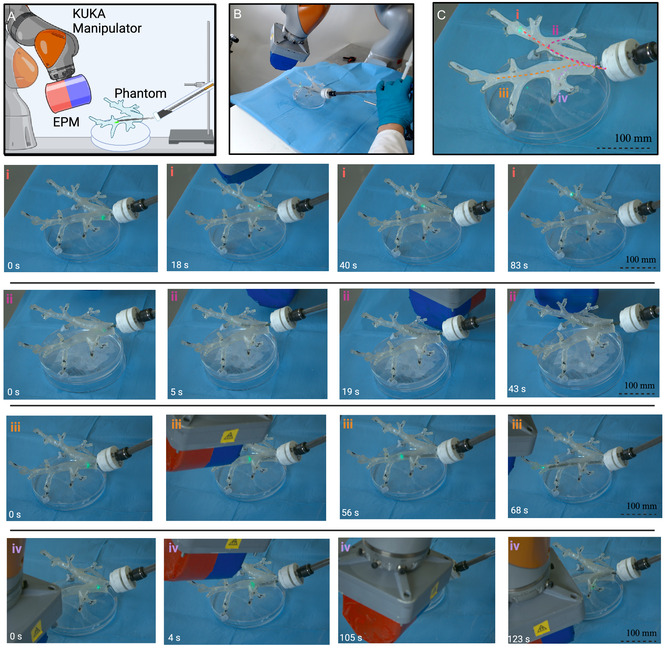
A) Diagram of experimental setup. The phantom is placed within the workspace of the manipulator and the vine robot introduced to the start point. The EPM is then manipulated using a gamepad to direct the vine robot through the phantom. B) Real‐world experimental setup. C) Diagram showing four target paths on the phantom.

To evaluate the ability to navigate through bifurcations, we utilized a 3D‐printed phantom of a bronchial tree (LIDC‐IDRI‐0807) (www.cancerimagingarchive.net). The vine robot (5 mm diameter) was positioned at the base of the trachea and the system was placed within the workspace of the manipulator and EPM (see **Figure** **9**). To track the position of the vine robot, a LED fiber was inserted into the tool channel and the duty cycling approach (as described in Section 2.3) was applied manually to maintain the fiber at the tip during growth. Four target areas of the bronchial tree were established and four repeats were performed. The robot manipulator was controlled via the joystick to position the EPM. The average time for the four navigations and path lengths is specified in **Table** **2**. Due to the magnetorheological effect, some small stiffening of the vine was found to inhibit growth at close proximity to the EPM. Therefore, the EPM had to be intermittently retracted to a greater distance to allow growth (see Video S3, Supporting Information). A detailed exploration of this phenomenon is beyond the scope of this work but is addressed in the conclusion.

**Table 2:** Table describing average times for the navigation experiment in Figure 9.

Navigation	Path length [mm]	Success rate	Mean time [s]	Standard deviation [s]
i	112	4/4	69	19
ii	99	4/4	109	14
iii	104	4/4	125	27
iv	61	4/4	110	30

### 4.5 Clinical Case Study: Peripheral Lung Biopsy

**Figure 10:**
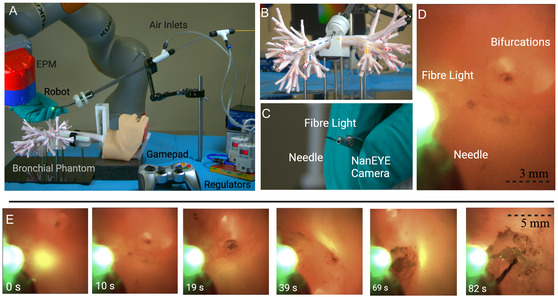
A) Real‐world experimental setup. B) Bronchial tree phantom. C) NanEYE camera mounted to fiber with a tissue biopsy needle. D) Features as seen by the internal camera. E) View from endoscopic camera in navigation (i), reaching a target tissue and penetration with the biopsy needle.

Finally, we demonstrate the use of the magnetic fluid‐driven vine robot (5 mm diameter) in a bronchoscopy training simulator for the application of tissue biopsy (Ultrasonic Bronchoscopy Simulator, Adam Rouilly, UK). We attach a NanEYE 1 mm × 1 mm RGB CMOS camera (AMS‐OSRAM, USA) to the end of the optical fiber and implement camera‐assisted navigation (see Video S4, Supporting Information) (**Figure** **10**B). The wire for the camera was kept external to the tool channel for simplicity of attachment, but in the future would be integrated into the vine tool channel. The same duty‐cycle approach was applied for keeping the illumination fiber at the tip, with the secondary pressure source utilized for further reduction of friction within the body. Two target areas were established and the EPM was manually positioned. The navigation was completed using the internal camera for approximate location and visual inspection of the phantom was used to correlate this with the real bifurcation. In a clinical setting, this step would be preformed with a preoperative CT scan of the lungs. The navigations were repeated four times with 100% success rate averaging 92 and 140 s respectively and standard deviations of 15 and 33 s. Figure 10E shows the first navigation, from the endoscopic camera. After reaching the target anatomy, the needle is inserted into a piece of soft phantom tissue, replicating a biopsy procedure. The ability to penetrate the target tissue is assisted by the vine robot's stabilization on the surrounding vessel.

## 5 Discussion and Conclusion

While the pressurization of everting structures allows them to locomote, the steering of miniaturized vine robots has proven challenging. A magnetic fluid used in lieu of air or water serves the simultaneous purposes of growing the vine under pressure while also enabling steering via deflection under magnetic fields. This novelty retains the entirely soft structure of the vine allowing squeezing through gaps and fully compliant behavior along the vine's length. The simplicity of the structure allows for the miniaturization of the device enabling bending in any arbitrary plane, given the EPM can be appropriately positioned. Via teleoperated control, under feedback from a tip‐mounted camera, the robot can accurately position a biopsy needle. The work of Li et al.^[^ ^27^ ^]^ observed that pressures up to 62 kPa did not visually damage any tissue in their bronchoscopy soft robot. Our demonstrated platform operates at pressures well below this value, with a maximum required growing pressure of 35 kPa. Further clinical studies of the safety of these pressures in the lungs will clearly be needed.

The functionalization via a biopsy needle shows a clinical use case for such robots, where the vine's ability to stabilize on surrounding lumens and apply large tip forces enables penetration of soft tissue. This, combined with the shear‐free movement and soft body of the robot, holds the potential for reduced traumatic tissue interaction compared to traditional tools. In the work of Van Lewen et al. to achieve a 1 N penetration force, a pressure of 200 kPa was needed which could potentially be dangerously high. Our work, via stabilization near the robot tip, is capable of a 1.26 N penetration force with a significantly (82%) lower pressure of 35 kPa.^[^ ^20^ ^]^ The ability to create these large tip forces is dependent on vine stabilization. Therefore, the vine robot diameter must be appropriate for the anatomy. Future work will consider how variable vine robot diameters can be digitally manufactured based on preoperative imaging, including tapered designs and hyperelastic ballooning vine robots capable of conforming to a wider range of lumen diameters.^[^ ^20^ ^]^

In our previous work, we adopted an approach based on a magnetic skin for the vine robot.^[^ ^18^ ^]^ This approach was constrained to very low magnetic volume thus limiting any induced magnetic wrench which, coupled with requisite growing pressures, limits achievable deformation. In this work, the use of a magnetic liquid gives a relatively high magnetic volume (over 95% by total volume of the robot) and therefore allows vines to be steered and grown simultaneously. The drawback to this approach is the magnetorheological effect which stiffens the fluid under high magnetic fields enforcing a balance between wrench and stiffening. While this characteristic could potentially be leveraged for a variable stiffness design, in the case of navigation (being discussed here), it is not helpful. A potential solution to this challenge would be the use of a magnetic fluid with low magnetorheological behavior. Approaches to this include the use of smaller magnetic particles (nanoparticles) whose chaining would likely be less significant on the bulk material properties. This would also come with the advantage of prolonged or permanent suspension of particles. With iron and water as the main ingredients, the iron suspension utilized is biocompatible; however, the effect of leakage of a large volume of iron into certain anatomies could potentially be dangerous. A current device used in clinical practice named EndoRail^[^ ^28^ ^]^ utilizes a similar iron–water suspension for assisting during colonoscopy procedures, further supporting the clinical viability of this technique. Future work should more closely consider the fluid of choice with respect to both magnetorheology and the risk of leakage pertaining to the specific procedure investigated.

The 5 mm robot demonstrated enables navigation up to the fourth generation of the bronchial tree^[^ ^29^ ^]^ but further miniaturization will be needed for deeper peripheral lung access. Our thermoplastic elastomer and laser sealing process enabled customizable vine diameters, essential for comparative studies, though the 3 mm design struggled with burst pressure limitations. Scaling down vine diameters increases the required growing pressure quadratically and therefore resistance to higher pressures is vital for miniaturization. Alternative approaches for forming cylindrical TPE structures include the heat pressing method of Rogatinski et al. (capable of holding pressures up to 300 kPa) and extrusion of thermoplastic elastomeric cylinders which would completely eliminate seam sealing.^[^ ^30^ ^]^ Miniaturization could also benefit from stronger polymers like LDPE or PTFE along with friction‐reducing lubricants or hydrophilic coatings.^[^ ^12^ ^]^ The magnetic fluid approach is inherently easier to implement at smaller diameters compared to other vine steering methods (as shown in Table 1) due to the lack of complex structures in the vine body. Further developments in vine wall material and construction techniques will allow the development of fully steerable vine robots at scales suitable for navigating tighter lumens.

The stiffening of the magnetorheological fluid was found in some cases to inhibit growth and therefore the EPM had to be withdrawn to allow growth. This stiffening could however be utilized for the application of large forces for stabilization and tissue interaction. Magnetorheological fluids have been explored for stiffening within soft robotic systems; however, they do rely on large magnetic fields^[^ ^31^ ^]^ (436 mT for a 344% change in stiffness). Other approaches to stiffening could also be explored such as granular or layer jamming; however, they have proved challenging at very small scales.^[^ ^32^ ^]^ Alternatively, low melting point alloys have been explored for vine stiffening and demonstrated at a 6.4 mm‐diameter scale^[^ ^33^ ^]^ achieving a 2100% stiffness change but requiring heating to 62 °C to achieve transition. This approach can of course result in the heating of surrounding tissue which has the potential to limit clinical viability.

When compared to pneumatically steered vine robots based on inbuilt pressurized pouches, our design provides a simple structure much more compatible with miniaturization requirements. Smaller vine robots have been presented based on steerable internal tendons; however, these sacrifice the vine's fully soft structure and the presence of complex internal mechanisms hinders further miniaturization. With the correct materials and manufacturing, magnetic fluid‐driven vines should be viable at a significantly smaller scale as long as the material is capable of eversion. Our work has focused on the control of the direction of growth of the vine robot; however, given the body‐steering methodology, it may be possible with localized field gradients to control the whole body enabling more complex deformation and shape control. Future work could consider applying this to compensate for movement in the lungs during respiration.^[^ ^34^ ^]^

Once a vine has navigated to the area of interest, its retraction must also be considered. The naive approach of simply retracting the tail leads to vine buckling for anything other than a straight vine. This is true of all soft vine robot methodologies independent of actuation approach.^[^ ^26^ ^]^ In our case, we removed the vine from the anatomy simply by deflating it to reduce its diameter and pulling it from the proximal end in a similar manner to a standard endoscopic tool. However, an ideal system would invert the vine in the opposite manner to the eversion process, enabling shear‐free movement for both insertion and retraction. Active retraction systems based on complex internal mechanisms that retract the vine material have been developed;^[^ ^35^ ^]^ however, applying systems such as these to highly miniaturized vines remains an open challenge. Others have considered the use of stiff internal tools to aid retraction; however, this sacrifices the soft body of the robot.^[^ ^7^, ^36^ ^]^ The use of magnetics for stabilization during retraction has also been explored; however, this approach currently relies on high magnetic volumes and high fields.^[^ ^19^ ^]^ Future work will consider retraction mechanisms suitable for magnetic fluid‐driven vines at this scale.

The minimal growing pressure for a vine robot depends on path‐independent properties (e.g., material, thickness, viscosity, and diameter) and path‐dependent properties (e.g., grown length and curvature).^[^ ^11^ ^]^ While this work uses a reliable experimentally determined growing pressure (Figure 5B), future studies will explore qualitative models, especially considering the influence of magnetic fields on fluid viscosity. Modeling the deflection of magnetic fluid‐driven vine robots is challenging due to complex interactions between the flexible pressurized walls, magnetic properties, fluid behavior, and the anatomy through which the robot travels. These effects are difficult to predict using current methods. The simple model presented in Figure 2 compares magnetic fluid with magnetic skin but neglects many complexities, such as demagnetization effects and fluid suspension behavior, focusing instead on maximal torque. Future research should develop detailed models of these phenomena. Combining these models with internal sensing will enable a closed‐loop control strategy, giving rise to more intuitive robotized systems.

The focus of this work has been on the exploration of a novel approach to vine steering, the design of suitable magnetic fluids, relevant hardware, and potential application in tissue biopsy procedures. The control approach has been manual with the manipulation of the EPM pose via a gamepad. Compared to other magnetic manipulation systems based on hard internal magnets, the use of magnetic fluids which instantly align with the magnetic field provides a relatively intuitive system to control via a gamepad. Nevertheless, due to the low magnetization of soft magnetic materials when compared to their magnetically hard counterparts, the required distance between EPM and internal robot continues to be smaller than a clinically ready system. Future work will consider either increasing the magnetic volume of the magnetic fluid or alternate magnetic manipulation systems capable of generating larger magnetic field gradients. The duty‐cycle approach adopted for holding tools at the tip was also operated manually. This should be automated as seen in previous work in order to improve the usability of the system, lowering the task burden and allowing growth to also be controlled via the gamepad.^[^ ^7^ ^]^ It would also be desirable to implement a closed‐loop control strategy within this framework. This could be approached via fiber‐Bragg shape sensors or by integrating magnetic field sensors into the robot,^[^ ^37^ ^]^ coupled with appropriate modeling of the vine and interactions with magnetic fields and the surrounding physical environment.^[^ ^38^ ^]^

Here, we have demonstrated the possibility of utilizing magnetic fluid for the steering actuation of miniaturized vine robots. We have demonstrated the functionality of these robots at diameter scales from 4 to 7 mm and explored their application in minimally invasive tissue biopsy procedures. Further miniaturization is dependent on the ability to form thin‐walled flexible tubes which can withstand higher pressures. This will provide new avenues for the application of magnetic fluid‐driven vines to ever smaller lumens where their distinct advantages of mechanical compliance and zero‐shear navigation can be further leveraged.

## Conflict of Interest

The authors declare no conflict of interest.

## Author Contributions

**Joshua Davy**: conceptualization (lead); data curation (lead); formal analysis (lead); investigation (lead); methodology (lead); project administration (lead); resources (lead); writing—original draft (lead); writing—review & editing (lead). **Thomas P. Dean**: data curation (supporting); formal analysis (supporting); methodology (supporting). **Nikita J. Greenidge**: conceptualization (supporting); writing—review & editing (supporting). **Benjamin Calmé**: conceptualization (supporting); supervision (supporting); writing—original draft (supporting). **Peter Lloyd**: supervision (supporting); writing—original draft (supporting). **James H. Chandler**: supervision (supporting); writing—original draft (supporting). **Pietro Valdastri**: conceptualization (supporting); funding acquisition (lead); supervision (lead); writing—original draft (supporting).

## Supporting information

Supplementary Material

## Figures and Tables

**Figure A1:**
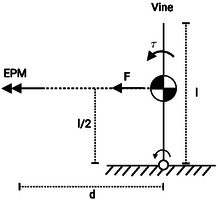
Diagram showing rigid body assumptions used in the mathematical analysis comparing magnetic skin and magnetic fluid approaches discussed in section.

**Figure A2:**
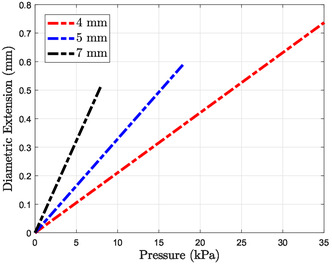
Diametric extension for various vine diameters up to reliable growing pressure as established in Section 4.1.

**Figure A3:**
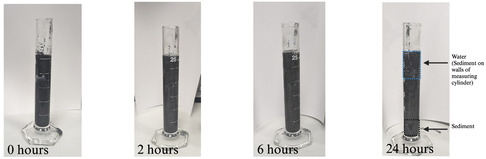
Settling of the iron particles in the magnetic fluid. The fluid remains usable for 0–12 h before agitation is needed. The fluid is visibly settled into layers after 24 h.

## Data Availability

The data that support the findings of this study are available from the corresponding author upon reasonable request.

## Appendix A1 Calculation of Magnetic Moment and Torques

### A1.1

For the case of magnetically hard skin, the magnetic moment when magnetized orthogonally to the main axis is calculated as

(A1)
$$\left|m_{s}|=|m_{\text{outer}}+m_{\text{inner}}|=|m_{\text{outer}}-0\right|$$

as given in our previous work^[^ ^18^ ^]^ where **m**_outer_ is the moment of the outer everted layer and **m**_inner_ = 0 is the moment of the inner tail, equal to zero in the case of orthogonal magnetization. Given the hard magnetic material utilized and an actuating field significantly below that of the saturation magnetization

(A2)
$$m_{s}=\frac{B_{r}V}{\mu_{0}}$$

where **B**_r_ is the remanent magnetic flux density vector. *μ*_0_ is the magnetic permeability of free space and *V* is the volume of the everted magnetic skin equal to 2*πrwl*, where r is the robot radius, *w* is the skin thickness, and *l* is the length of the vine. For the NdFeB‐doped Ecoflex‐30 silicone utilized in our previous work, $\left|B_{r}\right|$ is equal to 0.1 T.^[^ ^39^ ^]^

For the magnetic fluid approach, magnetic moment is a function of the applied field

(A3)
$$m_{f}=\sigma \chi HV$$

where *σ* is the volumetric proportion of magnetic fluid in this case equal to 0.23 as discussed in Section 2.2, *χ* is the volume magnetic susceptibility of iron equal to 4999 (dimensionless), and *V* is the volume of magnetic fluid in the vine equal to *πr* ^2^ *L*.

Maximal torques are calculated under rigid body assumptions with a pivot point at the vine base (see **Figure** **A1**). The vine is assumed to be positioned along the EPM major axis at a distance *d* and the fields and gradients are calculated under dipole assumption as given by the dipole model

(A4)
$$B(m_{e},r)=\left(\frac{\mu_{0}}{4\pi {\left\|r\right\|}^{3}}(3\hat{r}{\hat{r}}^{T}-I)\right)m_{e}$$

where *r* is the displacement between measurement and dipole position, **m**_e_ is the magnetic moment of the dipole, and $\hat{r}=\frac{r}{\left|r\right|}$, with $H=\frac{1}{\mu_{0}}B$ in free space.

Maximal torque can be calculated as the summation of the magnetic body torque (in the case of the hard magnetic skin) and the induced moment from the magnetic force acting on the pivot point equal to

(A5)
$$\tau_{\text{max}}=|\tau +F\frac{l}{2}|=|m\times B|+|{\left(\nabla B\right)}^{T}m|\frac{l}{2}$$

## Appendix A2 Modeling of Diametric Extension Under Pressure

### A2.1

The stress at the vine wall can be calculated under the hoop stress equation.^[^ ^40^ ^]^

(A6)
$$\sigma =\frac{Pd}{2t}=E\varepsilon$$

where *P* is the pressure, *d* is the vine diameter, *t* is the material thickness, *E* is the elastic modulus, *ε* is the strain, and *σ* is the stress. Plotting this for vine diameters of 4, 5, and 7 mm, we can calculate the diametric extension (see **Figure** **A2**). Pressures are plotted up to the reliable growing pressure established in Section 4.1 (Figure 5B).

It can be observed that the diametric extension is less than 1 mm for all reliable growing pressures.
